# Extracellular *α*-Galactosidase from *Trichoderma* sp. (WF-3): Optimization of Enzyme Production and Biochemical Characterization

**DOI:** 10.1155/2015/860343

**Published:** 2015-11-02

**Authors:** Aishwarya Singh Chauhan, Arunesh Kumar, Nikhat J. Siddiqi, B. Sharma

**Affiliations:** ^1^Department of Biochemistry, Faculty of Science, University of Allahabad, Allahabad 211002, India; ^2^Department of Biochemistry, SHIATS, Naini, Allahabad 211007, India; ^3^Department of Biochemistry, King Saud University, Riyadh 11495, Saudi Arabia

## Abstract

*Trichoderma* spp. have been reported earlier for their excellent capacity of secreting extracellular *α*-galactosidase. This communication focuses on the optimization of culture conditions for optimal production of enzyme and its characterization. The evaluation of the effects of different enzyme assay parameters such as stability, pH, temperature, substrate concentrations, and incubation time on enzyme activity has been made. The most suitable buffer for enzyme assay was found to be citrate phosphate buffer (50 mM, pH 6.0) for optimal enzyme activity. This enzyme was fairly stable at higher temperature as it exhibited 72% activity at 60°C. The enzyme when incubated at room temperature up to two hours did not show any significant loss in activity. It followed Michaelis-Menten curve and showed direct relationship with varying substrate concentrations. Higher substrate concentration was not inhibitory to enzyme activity. The apparent Michaelis-Menten constant (*K*_*m*_), maximum rate of reaction (*V*_max_), *K*_cat_, and catalytic efficiency values for this enzyme were calculated from the Lineweaver-Burk double reciprocal plot and were found to be 0.5 mM, 10 mM/s, 1.30 U mg^−1^, and 2.33 U mg^−1^ mM^−1^, respectively. This information would be helpful in understanding the biophysical and biochemical characteristics of extracellular *α*-galactosidase from other microbial sources.

## 1. Introduction

The *α*-galactosidase belongs to an important class of hydrolase enzymes with various applications based on its unique design for catalyzing hydrolysis of *α*-1,6-linked *α*-galactose residues from oligosaccharides such as melibiose, raffinose, stachyose, and galactomannans, as well as galactolipids. Transgalactosidase activity was also demonstrated in case of some *α*-galactosidases. Nowadays, *α*-galactosidases have an increasingly practical potential in biotechnology. *α*-galactosidase is widely distributed among plants, bacteria, and fungal system [1, 2], but humans and monogastric animals lack *α*-galactosidase in their digestive tract [3]. Among all the sources of *α*-galactosidase, the fungal *α*-galactosidases were most suitably exploited for their biotechnological applications mainly due to their extracellular localization, acidic pH optima, and broad stability profiles. Currently, different fungal enzymatic machineries are being utilized by many food, feed, pharma, and biotechnological industries for the synthesis of desired and differently cleaved products. Among them, many sources have been identified and purified from many* Aspergillus sp*.,* Trichoderma sp*.,* Penicillium sp.*, and so forth [4–11].

In order to improve the nutritional value of legume-based food, they can be applied for reduction or elimination of antinutritive galactooligosaccharides (called raffinose family sugars) that cause flatulence. Galactooligosaccharides produced by transfer reaction of *α*-galactosidase can be used as a prebiotic in functional food. In beet industry, they are used to remove raffinose from molasses in order to facilitate the crystallization and improve the yield of sucrose. Furthermore, in the pulp and paper industry, the use of hemicellulases, including *α*-galactosidase, has gained interest. Moreover, the potential of *α*-galactosidase can be exploited for medical purposes and as a biochemical tool in structure analysis. Enzyme replacement therapy with *α*-galactosidase is considered as a potential treatment for Fabry's patients [12]. In addition, the enzyme is applied for the conversion of type “B” erythrocytes to type “O” erythrocytes [13] and in xenotransplantation [14]. Currently, *α*-galactosidase is commercially available as dietary supplement in trade name of Beano [15] and so forth and as Fabrazyme for treatment of Fabry's disease in humans [12]. An elaborated survey of physiological role and applications of *α*-galactosidase is itself very interesting and wider area of research could be explored in near future.

The present study is an endeavor to obtain maximum production of extracellular enzyme from culture filtrate of* Trichoderma sp.* under* in vitro* enzyme assay conditions. Our investigation is based on isolation and examination of new fungal sources for *α*-galactosidase production. This paper presents efficacy of newly isolated* Trichoderma sp.* for its immense potential to produce good amount of *α*-galactosidase extracellular. Earlier reports from this laboratory have indicated that soybean flour was the best carbon substrate in secondary media for *α*-galactosidase production by* Trichoderma sp.*, which was possibly due to the presence of various suitable nutrients in soybean flour and/or due to its most suitable particle size and consistency required for anchorage, colonization, and enzyme secretion [16]. The biophysical and biochemical properties of this enzyme have also been determined here under different experimental conditions. The results of this study would be useful in understanding properties analogous enzymes from other fungal and bacterial sources and to exploit them for varied biotechnological, industrial, and clinical applications.

## 2. Materials and Methods

### 2.1. Chemicals

p-Nitrophenyl-*α*-D-galactopyranosides (pNPGal), synthetic substrate for screening of *α*-galactosidase activity, and para-nitrophenol (pNP), chromogenic substrate for standard preparation, were purchased from Sigma Chemical Co. (St. Louis, MO, USA); Folin-Ciocalteu's phenol reagent and sodium carbonate were from Merck Chemical Supplies (Darmstadt, Germany). All other chemicals used were of analytical grade.

### 2.2. Fungi

Selected fungi, that is,* Trichoderma sp.* (WF-3), were isolated from rhizospheric soil of* Phyllanthus emblica* (aanwla) collected from the local garden of Sagar, India, as discussed in previous paper [16]. They were isolated by direct soil plate method [17] and identified according to [18–20]. Pure cultures were maintained on PDA slants.

### 2.3. Culture Medium and Conditions

A basal solution consisted of KH_2_PO_4_ (7.0 g L^−1^), K_2_HPO_4_ (2.0 g L^−1^), MgSO_4_·7H_2_O (0.1 g L^−1^), (NH_4_)_2_SO_4_ (1.0 g L^−1^), yeast extract (0.6 g L^−1^) in tap water to a volume of 1000 mL, and 1% (w/v) dry contents of each of the selected substrates such as extracts of guar gum (GG), soybean meal (SM), and wheat straw (WS). The galactose and sucrose were added separately and in combination into the basal medium. For each particular treatment, 250 mL Erlenmeyer conical flasks were prepared containing 50 mL of basal or optimized media, sterilized at 121°C for 20 min under 1.5 atmospheric pressure and cooled to room temperature. On cooling, they were inoculated by addition of three pellets of heavily sporulated fungi from 4- to 5-day-old cultures that were picked up through cork borer (1 × 1 cm in diameter) and were added to Erlenmeyer flasks each of 250 mL capacity (105 spores mL^−1^, if otherwise not stated). The cultivation was carried out on rotary shaker (120 rpm) and incubated at 28°C for 11 days. The *α*-galactosidase activity and protein content of the culture filtrate were determined in the culture filtrates.

### 2.4. Enzyme Activity Assay

The *α*-galactosidase assay was carried out in test tubes by the modified version of the method by using p-nitrophenyl-*α*-D-galactopyranoside (pNPGal) as a substrate. The assay system contained 0.5 mL of 0.05 M sodium acetate buffer (pH 5.0), 0.9 mL of 1.0 mM pNPGal solution, and 100 *μ*L of enzyme preparation (culture filtrate). The reaction was started by addition of pNPGal. The reaction mixture was incubated for 10 min at 50°C and was stopped by the addition of 0.5 mL of 1.0 M sodium carbonate solution. The amount of p-nitrophenol (pNP) released was determined spectrophotometrically using UV-Visible double beam spectrophotometer (Spectrascan UV 2700) at 405 nm. One unit (U) of enzyme was defined as the amount of *α*-galactosidase enzyme which liberates 1 *μ*mol of pNP min^−1^ mL^−1^ under the given assay conditions.

### 2.5. Protein Estimation

The extracellular protein content excreted in the culture filtrate by each of the fungal strain was determined by the method as described [21]. The bovine serum albumin (BSA) was used as a standard. The culture filtrate without any fungal inoculums was used as a control from each set of experiments.

### 2.6. Determination of Effect of pH on Enzyme Activity

The enzyme activities were assayed at 50°C for 10 min. of incubation in water bath in different buffers like acetate, tris-glycine, citrate, citrate phosphate, and glycine NaOH of different pH values, respectively, ranging from pH 2.5 to pH 10. The enzyme activity profile prepared after assaying it at different pH values (Table 1) indicated that the enzyme was optimally active at a broad acidic range of pH from 5.0 to 6.0. The best buffer under enzyme assay conditions was citrate phosphate buffer of pH 6. The enzyme activity was, however, noticed to be stable between pH ranges from 5 to 7.

### 2.7. Effect of Temperature on Enzyme Activity and Stability under Assay Conditions

**Figure 1:**
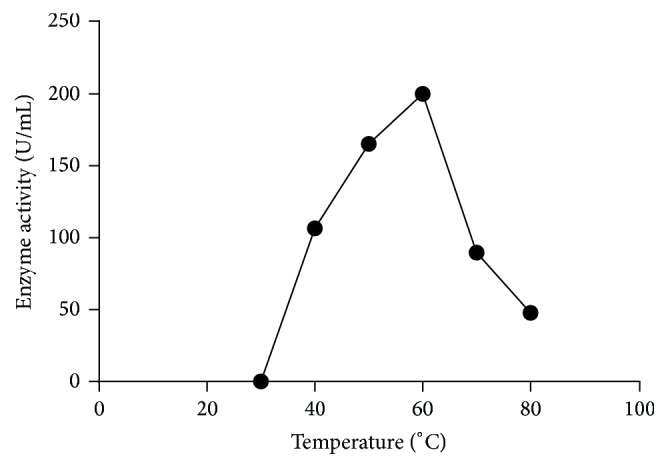
Effect of temperature on enzyme activity. The enzyme was assayed at different temperatures as described in Section 2.

The enzyme activities were assayed under the standard assay conditions at pH 5 for 10 min at various temperatures ranging from 30 to 80°C.  Figure 1 shows the temperature-activity profile of *α*-galactosidase. The enzyme showed maximum activity at 60°C.

### 2.8. Effect of Different Incubation Time on Enzyme Activity and Stability Profile under Assay Conditions

**Figure 2:**
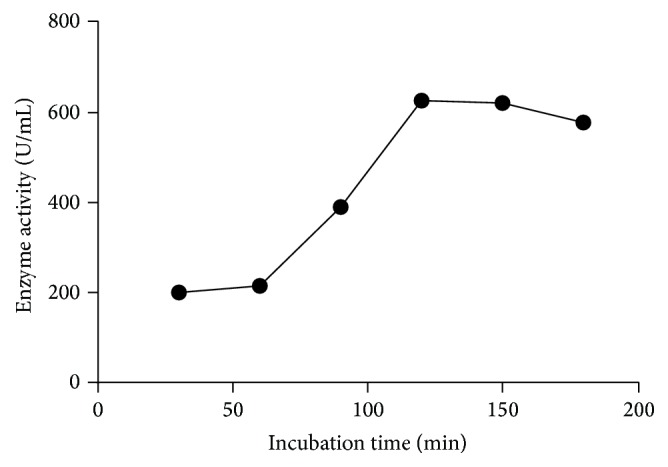
Effect of incubation time on enzyme activity. The effect of varying time on the enzyme activity when it was incubated at room temperature as described in Section 2.

In order to estimate the stability of enzyme for different intervals such as 15 min to 3 hr shown in Figure 2, the residual activity was assayed under standard conditions where incubation temperature kept constant that is at 60°C. Interestingly, the results showed greater stability of enzyme and increase in activity with increasing interval of incubation time under* in vitro* enzyme assay conditions.

### 2.9. Effect of Substrate Concentration on the Rate of Enzyme Catalyzed Reaction

**Figure 3:**
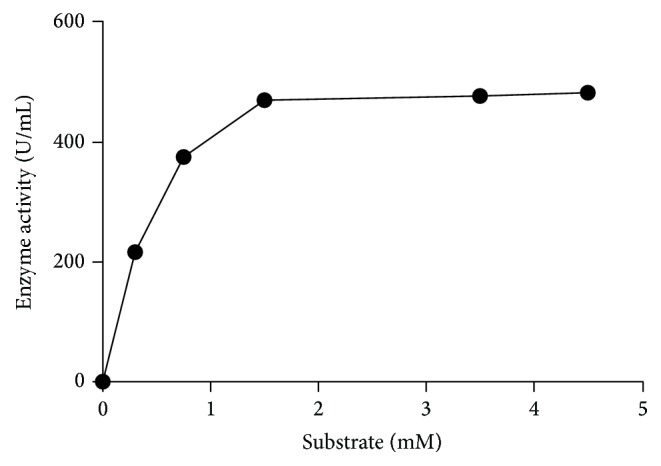
Effect of varying substrate concentrations under enzyme assay condition. The enzyme activity was monitored as described in Section 2. The enzyme follows simple Michaelis-Menten curve and a direct relationship between activity and substrate concentration.

**Figure 4:**
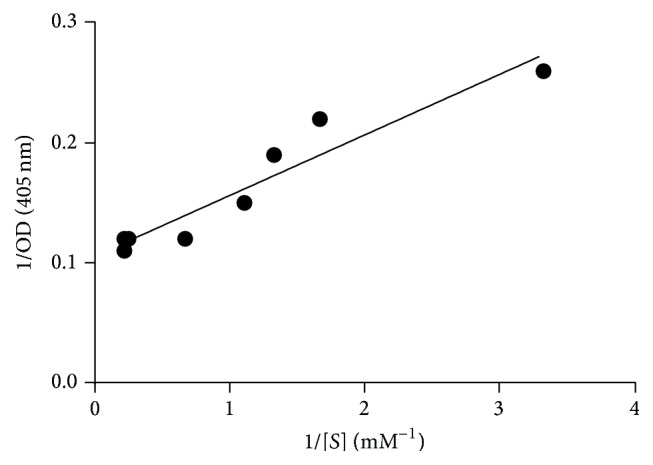
Lineweaver-Burk double reciprocal plot of the data from Figure 3. The *K*_*m*_, *V*_max_, and *K*_cat_ and catalytic efficiency values from the above plot have been calculated.

The effect of substrate concentration on the rate of hydrolysis of p-nitrophenyl-*α*-D-galactopyranoside was investigated. Using varying concentrations of p-nitrophenyl-*α*-D-galactopyranoside from 0.05 to 4.5 mM, when the enzyme assay was done following the standard assay procedure, it followed simple Michaelis-Menten kinetics. The apparent Michaelis-Menten constant (*K*_*m*_), maximum velocity (*V*_max_), *K*_cat_, and catalytic efficiency values for the enzyme were calculated from the Lineweaver-Burk plot shown in Figures 3 and 4.

### 2.10. Statistical Analysis of Data

Statistical analysis of data was performed using GraphPad Prism version 6 for windows. All values were expressed as mean standard deviation of 3 different observations.

## 3. Results and Discussion

The *α*-galactosidase activity was detected for a number of microorganisms. However, purification to homogeneity and characterization of this enzyme have only been done for a few organisms [22]. Characterization of any enzyme is of paramount importance in revealing the novel biochemical and catalytic properties suitable for its excellent industrial application. The enzyme recovery process is considered to begin once the fermentation has achieved peak yield. A commercial enzyme should be stable and fast-reacting during reaction, should have low transferase activity, and should be produced by organisms free of toxicity. Reports are available in the literature documenting production and characterization of different microbial strains. Cloning and expression of the gene encoding *α*-galactosidase in* S. coelicolor* A3(2) [23] have been done. Crystal structure of *α*-galactosidase from* Trichoderma reesei* [24] and rice [25] has been elucidated. Competitive inhibition by galactose is a common characteristic of most of the *α*-galactosidases [26, 27].

The results from the present paper indicated that* Trichoderma sp.* exhibited tremendous potential to secrete *α*-galactosidases extracellularly under certain optimized culture conditions [16].* Trichoderma sp.* have been used widely in food fermentation and secretion of degrading enzymes and biocontrol activities [28–30]. They are generally recognized as safe by many investigators; nevertheless, detailed toxicity studies must be conducted before a final conclusion is drawn.

### 3.1. Effect of Different pH Systems on the Enzyme Activity

The pH optimum of the* Trichoderma sp.* *α*-galactosidase is determined with different buffer systems like acetate, tris-glycine, citrate, citrate phosphate, and gly (NaOH) of different pH ranging from pH 2.5 to pH 10. For estimation of the effect of pH on *α*-galactosidase activity, reaction mixture contains 0.2 mL of buffer solution of different pH values along with substrate. The maximum enzyme activity was obtained at pH 6 and the most suitable buffer was citrate phosphate buffer of pH 6 followed by citrate buffer of pH 5 (Table 1). Most of the *α*-galactosidases are stable over a broad range of acidity. Generally, bacterial *α*-galactosidase has a pH optimum in the range of 6.0 to 7.5 [31, 32], while the pH optimum of the fungal and yeast *α*-galactosidase is about 3.5 to 5.0 [24, 33].

### 3.2. Effect of Temperature on Enzyme Activity and Stability under Assay Conditions

The temperature stability of the enzyme is based on the culture habits of microorganisms. Obviously, exhibited temperature stability is because the fungus is mesophilic in nature. This enzyme is fairly heat stable. Maximum residual enzyme activity was at 60°C. Inactivation occurs at temperatures higher than 60°C (Figure 1). The properties of the *α*-galactosidase from* Trichoderma sp.* and other sources are comparable. In earlier reports, *α*-galactosidase from* Pycnoporus cinnabarinus* has an optimum reaction temperature of 75°C and is also stable at this temperature [33]. The optimum temperature for most of the *α*-galactosidases lies in the range of 37 to 40°C [24].

### 3.3. Effect of Different Incubation Time on Enzyme Activity and Stability Profile under Assay Conditions

In order to investigate the effect of incubation time on enzyme activity where enzyme exhibits maximum turn over number, different incubation periods (15, 30, 60, 120, 150, and 180 min) were employed under standard assay conditions. While keeping incubation temperature constant that is 50°C, the enzyme activity gradually increased with each course of time and attained its maximum value at 120 min of incubation followed by 60 min (Figure 2).

### 3.4. Effect of Substrate Concentration on Enzyme Activity under Standard Enzyme Assay Conditions

Most of the microbial *α*-galactosidases have in common the fact that they can hydrolyze the synthetic substrates more extensively than the natural *α*-galactosidases [7]. To develop any enzyme-based process, knowledge about the kinetic parameters of the enzyme is of utmost importance. For this enzyme in the present study, the values for kinetic constants such as *V*_max_, apparent Michaelis-Menten constant (*K*_*m*_), *K*_cat_, and catalytic efficiency have been determined from the Lineweaver-Burk double reciprocal plot and have been found to be 0.5 mM, 10 mM/s, 1.30 U mg^−1^, and 2.33 U mg^−1^ mM^−1^, respectively, as shown in Figures 3 and 4. No significant inhibition in enzyme activity was observed due to higher substrate concentrations present in the assay mixture. The synthetic substrates are much more popular than complex substrates for defining *K*_*m*_ and *V*_max_ as they are convenient [34, 35]. Various natural substrates like raffinose, melibiose, stachyose, and so forth and synthetic substrates such as *ρ*N*ρ*-*α*-D-galactopyranoside are used for determining kinetic parameters for alpha-galactosidases. For an *α*-galactosidase from* Escherichia coli,* the *K*_*m*_ value was 0.12 for *ρ*-nitrophenyl-alpha-D-galactoside and 2.33 and 3.65 for melibiose and raffinose [32].

## 4. Conclusion

*Trichoderma* sp. used in the present investigation displayed significant potential to excrete or secrete *α*-galactosidase in the culture filtrate. This enzyme exhibited its preference for larger acidic range of pH specificity for its optimum activity. It also showed selectivity towards a specific temperature to be optimally active in the extracellular milieu. The enzyme proved to be highly stable at room temperature up to two hours without losing any significant amount of activity. It followed Michaelis-Menten curve and showed perfect relationship with varying substrate concentrations. It could not get inhibited by higher substrate concentration. For this enzyme, the apparent Michaelis-Menten constant (*K*_*m*_), maximum rate of reaction (*V*_max_), *K*_cat_, and catalytic efficiency values for this enzyme as calculated from the Lineweaver-Burk double reciprocal plot were 0.5 mM, 10 mM/s, 1.30 U mg^−1^, and 2.33 U mg^−1^ mM^−1^, respectively. This information may help in understanding the catalytic behaviour related to enzyme, substrate, and environment specificity. This information on *α*-galactosidase may be useful in developing insight into the biophysical and biochemical characteristics of analogous extracellular *α*-galactosidases from many other microbial sources and their possible industrial/clinical applications.

## Figures and Tables

**Table 1:** Effect of different pH (*in vitro*) on assay condition of *α*-galactosidase activity (U/mL).

Buffer	pH	Enzyme activity at the 5th day of culture incubation	Enzyme activity at the 6th day of culture incubation
Citrate	3	4.221 ± 0.92	39.66 ± 2.42
	4.6	4.470 ± 0.88	172.85 ± 8.31

Tris(glycine)	3	2.603 ± 0.72	21.11 ± 1.23
		3.130 ± 0.33	16.34 ± 0.98

Citrate phosphate	5	2.980 ± 0.22	48.80 ± 3.90
	6	5.578 ± 0.45	176.73 ± 9.12
	6.6	5.141 ± 0.42	57.56 ± 4.92
	7.4	5.540 ± 0.43	45.98 ± 3.92

Glycine (NaOH)	9.6	2.764 ± 0.32	122.43 ± 11.93
	10	2.603 ± 0.22	112.46 ± 10.77
